# Authors’ Response to Peer Reviews of “The Impact of Rural Alimentation on the Motivation and Retention of Indigenous Community Health Workers in India: Qualitative Study”

**DOI:** 10.2196/70059

**Published:** 2025-01-23

**Authors:** Ajit Kerketta, Raghavendra A N

**Affiliations:** 1 CHRIST (Deemed to be University), Hosur Road, Bhavani Nagar, Bengaluru, 560029, India, 91 8867055238

**Keywords:** rural alimentation, community health workers, motivation, retention, rural health, rural nutrition, workforce

*This is the authors’ response to peer-review reports for “The Impact of Rural Alimentation on the Motivation and Retention of Indigenous Community Health Workers in India: Qualitative Study.”*

## Round 1 Review [1]

### General Comments

*This paper [* 2 *] has given the impression that the researcher has done thorough homework before starting the research and it is evident in the paper. Case methodology and thematic analysis are a few of the approaches that depict the quality of the paper. Overall, as a reviewer, it is my opinion that the research paper is of quality.*

### Specific Comments

*1. A few more factors like government initiatives should be included in studying the impact on the motivation and retention of community health workers.*

Response: Factors such as government initiatives and policies have been additionally incorporated into the Discussion section.

### Major Comments

*1. I feel that the analysis also can include education as a parameter.*

*2. The thematic analysis is one of the strengths of this research and is appreciated.*

Response: Due to time constraints, education could not be included as a sample parameter.

### Minor Comments

*1. Common wording should be used in every section of the paper, like qualitative case research methodology and qualitative case research.*

Response: The term “qualitative case research” has been consistently used throughout the study.
